# Exploring Emotional Conflicts and Pain Experience in Patients with Non-Specific Chronic Neck Pain: A Qualitative Study

**DOI:** 10.3390/jcm14134748

**Published:** 2025-07-04

**Authors:** Yolanda Pérez-Martín, Milagros Pérez-Muñoz, Beatriz Martín-Castro, Susana Nunez-Nagy, Belén Díaz-Pulido, Isabel Rodríguez-Costa

**Affiliations:** 1 HIPATIA Research Group, Department of Nursing and Physical Therapy, Universidad de Alcalá, Ctra. Madrid-Barcelona (N-II), Km. 33,600, 28871 Alcalá de Henares, Madrid, Spain; milagros.perez@uah.es (M.P.-M.); belen.diazp@uah.es (B.D.-P.); isabel.rodriguezc@uah.es (I.R.-C.); 2 Physical Therapy Service, Miguel de Cervantes Health Care Center, Av. Gustavo Adolfo Bécquer, 23, 28806 Alcalá de Henares, Madrid, Spain; 3 Physical Therapy Service, Reyes Magos Health Care Center, Av. de los Reyes Magos, s/n, 28806 Alcalá de Henares, Madrid, Spain; beatriz.martin@salud.madrid.org

**Keywords:** neck pain, chronic pain, pain perception, mental health, psychological stress, emotional conflict, sociological factors, biopsychosocial model

## Abstract

**Background/Objective**: Non-specific chronic neck pain (CNP) greatly affects the social dynamics, the work performance, and the personal independence of patients. Research emphasizes the significant role of sociological factors, psychological stress, and emotional conflicts in the development, regulation, and endurance of chronic pain. This study aims to explore the influence of emotional conflicts on pain experience among CNP patients, drawing from their experiences. **Methods**: A phenomenological investigation was conducted, grounded in Heideggerian philosophy, involving CNP patients and healthcare professionals in Madrid, Spain. Participants were recruited from Primary Health Care centers. Data collection methods included semi-structured in-depth interviews, focus groups with patients, focus groups with healthcare providers, participant observation, and reflective diaries. Hermeneutic phenomenology guided the data interpretation. Thematic analysis was applied to transcribed audio recordings. **Results**: This study included 12 patients with CNP who participated in two in-depth interviews conducted at different time points—before and after receiving physiotherapy treatment. Additionally, 23 CNP patients took part in four focus groups, and 46 healthcare professionals (including physicians, nurses, and physiotherapists) participated in three focus groups. A hermeneutic analysis revealed the following three main categories: “Self-concept and pain experience”, “Daily life obligations and pain perception”, and “Emotional conflicts related to CNP”. Patients described themselves as nervous, having communication difficulties, and often prioritizing family or work tasks, leading to stress. They indicated that their interpersonal conflicts with close relations intensified their perceived pain in the neck, head, shoulders, and arms. **Conclusions**: From the perspective of the participants in this study, interpersonal and emotional conflicts appear to influence their perception of CNP.

## 1. Introduction

In 2020, the International Association for the Study of Pain provided a definition of pain as “an unpleasant sensory and emotional experience associated with, or resembling that associated with, actual or potential tissue damage” [1]. Pain is recognized as a multidimensional phenomenon, with dynamic interactions among biological, psychological, and social factors, each reciprocally influencing the others [2]. Moreover, climatic factors, such as temperature and atmospheric pressure, have been shown to influence referral patterns, further complicating the clinical presentation [3].

Chronic pain (CP) is defined by its persistence beyond the usual healing period, typically lasting for more than three to six months, depending on the clinical or research context. [1]. It is associated with anatomical and pathophysiological alterations, including peripheral and central sensitization, the formation of new neural connections, and pathology-specific brain alterations [4]. Psychosocial factors play a significant role in both the development and perpetuation of these changes. Personality, cognition, beliefs, learning, poor coping mechanisms, catastrophizing, depression, anxiety, post-traumatic stress, and emotional reactivity contribute to this complex interplay [2,5]. Negative emotions have been observed to activate various brain regions associated with pain processing, potentially exacerbating inflammatory responses and amplifying pain perception [6,7]. Furthermore, unconscious emotional reactions may heighten the perception of CP [7]. Sociocultural factors, such as inadequate social support, are also implicated in CP [2]. Research has suggested that interactions with significant others, including spouses, family members, and friends, are crucial for the adaptation to CP. Stressful or conflictual interactions may contribute to increased pain intensity and negative effects [8,9]. In this context, attachment styles have emerged as a key modulator of pain-related outcomes. Insecure attachment styles, such as anxiousness or avoidance, are associated with poorer pain outcomes and slower recovery [10], likely due to maladaptive threat responses shaped by early attachment experiences [11]. These patterns are also linked to catastrophizing, psychological inflexibility, and dysfunctional coping strategies [12,13]. By contrast, secure attachment fosters self-compassion and supports adaptive pain regulation [14].

Conversely, positive social relationships and emotions activate the neural pathways associated with adaptive responses, and may reduce pain intensity by modulating nervous system responses to stress and pain [15,16,17]. CP significantly impacts psychological and social well-being, often resulting in depression, anxiety, sleep disturbances, adverse social circumstances, reduced work capacity, impaired relationships, diminished self-esteem, increased risk of substance abuse, and decreased life expectancy [2,18,19,20]. Treatment protocols integrating pain education, emotional awareness, expression therapy, emotional and social skills training, and exercise have demonstrated efficacy in reducing pain intensity and pain interference in the short and medium term [21,22,23,24,25].

Chronic primary pain, characterized by functional impairment or emotional distress not attributable to another cause, is recognized as a distinct entity. It is often associated with significant emotional distress and functional disability disproportionate to any observable injury or disease, and it is considered a disease in itself [26]. Nociplastic pain mechanisms may underlie chronic primary pain [4]. Sociological factors, psychological stress, and emotional conflicts have been identified as influential factors in the generation, modulation, and persistence of chronic primary pain [27,28]. It is closely linked to social and interpersonal processes, including hypersensitivity to interpersonal rejection and difficulties in emotional awareness and expression [29,30,31].

Chronic primary musculoskeletal pain encompasses chronic primary pain affecting the muscles, bones, joints, or tendons [26], with non-specific chronic neck pain (CNP) falling within this category. CNP ranks as the fourth leading cause of years lost to disability, following back pain, depression, and arthralgia [27,32].

The American Psychological Association defines emotional conflict as a state of disharmony between intense, incompatible emotions, causing distress to the individual. Such conflicts often arise in close social relationships, leading to feelings of rejection or isolation, which contrast with the expected feelings of respect and support. Social pain (SP) refers to the experience of pain resulting from interpersonal rejection or loss, encompassing feelings of isolation, loss, rejection, and negative social feedback [33,34,35]. Imaging-based studies have identified the shared underlying neural pathways involved in processing SP and the affective dimension of physical pain [36,37,38,39]. Individual differences in sensitivity to SP correlate with sensitivity to physical pain, with factors modulating SP similarly affecting physical pain perception [37]. SP may be re-experienced long after the initial triggering event, and may be projected into the future, amplifying existing pain in the present [40,41].

There is a strong relationship between stress and CP perception [42,43,44]. Individuals with CP often exhibit higher perceived chronic stress and elevated cortisol levels [45]. Animal models have suggested that CP induces depressive symptoms, potentially explaining the co-occurrence of chronic stress, pain, and depression in humans [46]. Emotional conflicts induce psychological distress in individuals, influencing perceived pain. The impact of psychological stress on pain perception may be modulated through distinct biological mechanisms, including increased sympathetic nervous system activity, or heightened inflammatory responses [47]. Sustained stress may lead to continuous muscle contraction, resulting in microtraumas and the release of pain-inducing and inflammatory substances, thereby contributing to musculoskeletal alterations and pain [48].

Despite the existing evidence, many questions remain about how emotional and social life impacts pain perception in general—and CNP in particular. There is a lack of qualitative research exploring how the emotional experiences of CP patients influence their pain perception. This study offers a depth of understanding that quantitative data alone cannot provide, by uncovering the lived experiences of the participants that have often been overlooked in the previous research.

This study aims to explore the impact of emotional conflicts on pain perception in patients with CNP, using patient-reported experiences as the primary data source. Hermeneutic phenomenology offers a valuable and insightful approach for exploring the lived experiences of adults with CNP in depth. These findings could assist the development of comprehensive care strategies grounded in a biopsychosocial model.

## 2. Materials and Methods

### 2.1. Design

Qualitative phenomenological research was conducted to deeply explore the phenomenon. This study followed a qualitative approach rooted in hermeneutic phenomenology, guided by the philosophical perspective of Martin Heidegger. Heidegger proposed hermeneutics as a means of exploring and interpreting what it means to be human [49]. In this context, understanding emerges through the participant’s narrative, which offers access to their lived experience [50]. Attending to these personal accounts is crucial for deepening our understanding of individuals in healthcare settings, particularly those living with CNP. This study was conducted in accordance with the guidelines of the Declaration of Helsinki and was approved by Hospital Príncipe de Asturias Clinical Research Ethics Committee, Alcalá de Henares, Madrid (code OE 21/2015).

### 2.2. Context and Setting

This study was conducted between 2015 and 2018 at the Primary Health Care Centers (HC) and the University of Alcalá. Research was carried out in three HC centers situated in Alcalá de Henares under the Eastern Area Primary Health Care Management of Madrid, Spain. These 3 HC centers (Reyes Magos HC, Nuestra Sª del Pilar HC, and Juan de Austria HC) covered 11 basic health areas and served a population of 193,751 inhabitants in 2018.

### 2.3. Research Team

The research team comprised three physical therapists, who are also professors in the Department of Nursing and Physical Therapy at the University of Alcalá. All team members possessed clinical expertise in CP and prior research experience. Two team members specialized in qualitative research, another facilitated access to research sites, and the principal investigator conducted in-depth interviews and moderated the groups. The principal investigator had prior experience working with individuals living with CNP, which informed the initial understandings of the phenomenon. These pre-understandings were acknowledged and reflected upon throughout this study, as part of the interpretive engagement with the data. The entire team participated in observation, coding, reflection, consensus building, and final report preparation.

### 2.4. Participants

The participants included 23 adults diagnosed with CNP, referred to the Primary Care Physical Therapy Units of three HC centers, and 46 primary care professionals responsible for providing health care to the CNP patients (including 16 physicians, 26 nurses, and 4 physical therapists).

The participants with CNP included in this study met the following criteria: they had been diagnosed with CNP by their physicians and has been referred to the Physical Therapy Units, were proficient in Spanish, and voluntarily agreed to participate by signing an informed consent form. Exclusion criteria included acute or subacute neck pain, recent surgery, neurological or cardiac conditions, pain resulting from specific injuries or diseases, language barriers, and active psychiatric disorders.

All physicians, nurses, and physical therapists from the three participating HC centers were invited to take part in this study. Those who agreed to participate and provided informed consent were included, totaling 46 professionals. All healthcare providers were regular staff members at the HC centers and had extensive experience in managing CP. They were responsible for attending to the CNP patients included in this study.

The physical therapists at the HC centers—one of whom was a member of the research team—facilitated contact with the management of the HC centers, which enabled access to both the patients and the healthcare professionals.

### 2.5. Recruitment and Sampling

Patients with CNP were recruited by the physical therapists. Once the eligibility criteria were confirmed and the patients were informed about the purpose of the study, a team researcher contacted those who expressed interest in participating. The principal investigator explained the study procedures, provided an information sheet, and presented the informed consent form for signature.

A total of 23 patients with CNP were included in this study. Of these, 12 participated in two in-depth, semi-structured interviews—one conducted prior to the initiation of physiotherapy treatment and the other immediately after its completion. All 23 patients took part in one of four focus groups: two held at the Nuestra Sª del Pilar HC Center (5 participants each), one at the Reyes Magos HC Center (8 participants), and one at the Juan de Austria HC Center (5 participants).

Primary care professionals from each HC center were recruited during team meetings, in which the study was introduced and the nature of their participation explained. Those who expressed interest were assigned to the focus group corresponding to their HC centers and scheduled for a session. A total of three focus groups were conducted, one at each participating HC center, involving a total of 46 healthcare professionals.

Theoretical and purposive sampling methods were employed to ensure diversity among the patients with CNP. All healthcare professionals who wished to participate were included.

### 2.6. Data Collection Methods

Data collection included 24 in-depth, semi-structured individual interviews with 12 patients with CNP, conducted both before and after the onset of physiotherapy treatment, as well as four focus groups with CNP patients and three with primary care professionals. Interviews and focus groups were conducted until data saturation was reached.

The interviews were conducted in a private setting, specifically in the physical therapist’s consultation room at the HC center. The interviews, each lasting between 50 and 60 min, were conducted by the principal investigator, a trained research team member with expertise in motivational interviewing. Active listening was prioritized, and flexibility was maintained to adapt the interaction to the participants’ responses, while using a pre-established interview guide that included questions about their pain experiences, daily life implications, pain modulators, self-perceptions, values, stressors, and emotionally charged conflicts perceived to affect their pain. To investigate the bodily responses emerging during the verbal expression of conflict—understood as manifestations of the emotional tension elicited—the participants were guided to mentally revisit personal conflicts and reconnect with the emotions involved. The participants were instructed to mentally relive personal conflicts and experience the associated emotions. Once this task was completed, they were asked to observe and describe any bodily sensations that arose, as well as to identify the specific areas of the body where these sensations were experienced. This methodology is grounded in the existing literature indicating that recalling personal conflicts may trigger SP [40], induce mental stress that can affect the perception of bodily sensations [44], and increase muscle tension [48].

The CNP patient focus groups were conducted in the HC center meeting room, and were facilitated by the same researcher who had conducted the interviews. Another team member was present as an observer, taking notes on individual contributions, accompanying nonverbal language, evolving participant attitudes, and group dynamics. The topics discussed included pain-related aspects, accompanying symptoms, consequences in daily life, pain-influencing factors, stressors that exacerbate pain, the role of relationships, the interplay between emotional and physical pain, and treatment options.

Focus groups with healthcare professionals were also conducted in the HC center meeting rooms, facilitated by the same interviewer and accompanied by another researcher as the participant observer. The topics included the characteristics of CNP patients, diagnosis, treatment, and educational interventions, as well as the impact of pain, contributing factors, emotional–physical pain interactions, cognitive processes, emotions, associated behaviors, and patient expectations.

The guides used for interviews and focus groups can be found in Appendix A (Table A1, Table A2, Table A3 and Table A4). These guides were applied in a flexible and adaptive manner, allowing the interviewer and facilitator to explore emerging themes and follow the participants’ narratives, while maintaining consistency in data collection.

All interviews and focus group sessions were audio-recorded and transcribed verbatim. Data collection was further enriched through participant observation and the reflective journal kept by the principal investigator. Additional variables were assessed to better describe the sample, including age, gender, educational background, employment status, anxiety and/or depression (Goldberg Anxiety and Depression Scale), and pain intensity (Visual Analogue Scale).

### 2.7. Data Analysis

Data were interpreted using a hermeneutic phenomenological approach. Before be-ginning the analysis, it was essential to reflect on any assumptions or biases related to the phenomenon. Since it is not possible to separate one’s self entirely from prior experiences, acknowledging and articulating pre-understandings, such as previous knowledge, assumptions, or personal perspectives, was key to ensuring a valid interpretation. Through the literature review, the principal investigator became increasingly aware of the barriers faced by adults living with CNP. As the interviews progressed, ongoing contact with the participants deepened her understanding of their condition. While previous experiences might be viewed as a potential limitation, they are inevitably shaped by the context in which we live. Making these experiences explicit adds transparency and enhances interpretive credibility.

Data analysis followed the procedure outlined by Leonard (1989) [51] as adapted by Fernández-Sola (2019) [52]:

(1) Thematic analysis: each transcript was read several times in a reflective process aimed at deepening the engagement with the participants’ stories [53]. This practice of dwelling helped to prevent superficial interpretations. Reflexive journaling and repeated readings of the transcripts supported the researcher in recognizing how their own perspective shaped the interpretation. Rather than attempting to set aside personal assumptions, the researcher engaged in a continuous process of self-reflection in line with Heidegger’s concept of the hermeneutic circle. Data were coded and general categories were identified as part of the final interpretive effort.
(2) Specific episodes were analyzed by re-examining all aspects of the particular situations, including the quotations within each category.
(3) Paradigmatic cases were sought, identifying the examples that captured the essence of a situation in a particularly illustrative way.

### 2.8. Credibility, Rigor, and Neutrality

This study employed various strategies to ensure credibility, rigor, and neutrality, including prolonged fieldwork, persistent observation, expert judgment, referential adequacy, accurate transcription, time triangulation, and researcher triangulation. A detailed description of the participants’ experiences, their environment, and the context of data collection was provided. To support the analytical rigor, the transcripts were shared with an interpretive team consisting of two PhD-trained physiotherapists with expertise in hermeneutic phenomenology. Team members independently reviewed the transcripts, identifying recurring patterns through the hermeneutic circle. The team then met to discuss individual interpretations, exploring similarities and differences until reaching a consensus. This iterative process reinforced the depth and trustworthiness of the findings.

## 3. Results

Twenty-three subjects with CNP, and forty-six healthcare professionals, including physicians, nurses, and physical therapists, participated in this study. All participants completed the study. A description of the participants using sociodemographic and clinical data is shown in Table 1.

**Figure 1:**
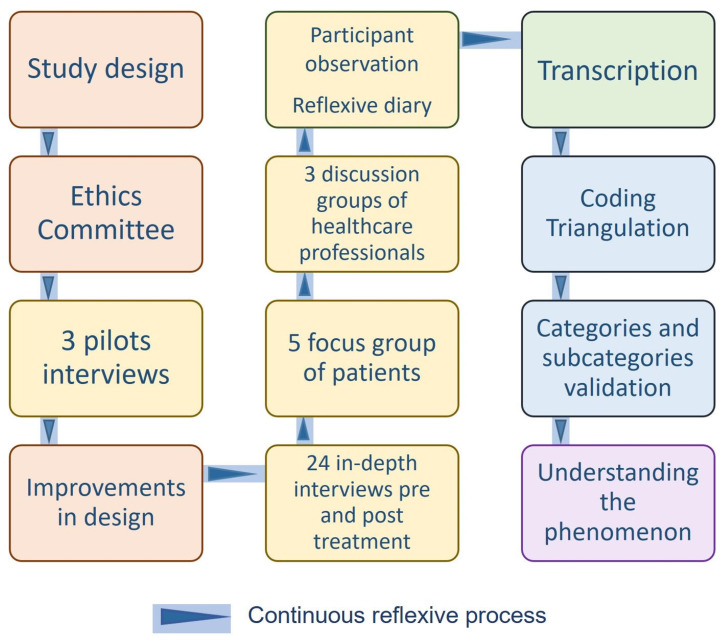
Research process flowchart.

Thirty hours of recordings were included from 24 in-depth interviews of 12 subjects with CNP (one before treatment started and the second one a month later), four focus groups with CNP patients (23 subjects), and three focus groups with healthcare professionals (46 subjects). Notes from the participant observation and reflective diary were also considered. Theoretical saturation was reached. The research process flowchart can be consulted in Figure 1.

The hermeneutic analysis revealed three overarching themes, referred to as primary categories, as follows: Self-concept and the experience of pain; Obligations of daily life and the perception of pain; Emotional conflicts related to CNP. These categories are elaborated upon in the subsequent sections.

The researcher’s prior understanding initially guided the interpretation but was later challenged and expanded through the participants’ narratives. Insights gained from reflexive engagement with the data allowed for a deeper appreciation of the nuances in the participants’ experiences.

### 3.1. Self-Concept and the Experience of Pain

This category delves into the personal characteristics shared by most CNP patients in this study, which significantly influenced their perceptions, interpretations, and actions, including their experience of pain [5]. It encompasses three subcategories as follows: Beliefs about themselves; Personal values; Personality traits.

#### 3.1.1. Beliefs About Themselves

Participants commonly viewed themselves as nervous, perfectionists, and having communication issues. These self-perceptions often impacted their interactions with others, their daily life management, and their coping with CNP.

“I get nervous about anything.”

(Participant 5/1st interview).

“I’m very perfectionist, so I want everything to go very well.”

(Participant 8/2nd interview).

“I’m not a conversation person either.”

(Participant 4/1st interview).

“These things (worries) you can’t tell a lot of people… Yes, it is just that to find a friend today… Where are the ideal friends? … There are very few people.”

(Participant 1/1st interview).

#### 3.1.2. Personal Values

This subcategory outlines the significant norms and customs guiding the participants’ actions and decisions. The key values expressed included independence, family, work, and sexuality.

“To be able to stand on my own two feet … not having to depend on anyone.”

(Participant 1/1st interview).

“First and foremost, my children, then my husband, then me (on a list of values).”

(Participant 3/1st interview).

“At work I am committed, serious above all.”

(Participant 7/1st interview).

“On a list of values, sexual ability I would put at the top of the list of what is fundamental.”

(Participant 12/1st interview).

#### 3.1.3. Personality Traits

Described aspects of personality limiting the individuals’ coping abilities and way of living. Commonly mentioned traits included self-devaluation and the need for expression and understanding.

“I’ve always had a bit of a complex about not achieving the level I should have had…”

(Participant 11/1st interview).

“We don’t worry about ourselves…, we don’t put our needs first…—First the children, first the husband, first the grandchildren…—We feel unappreciated, we have felt poorly valued.”

(Focus group with patients/Reyes Magos HC Center).

“Something happens to me, sometimes I don’t express what I feel, and when I don’t express myself, I feel bad…”

(Participant 6/1st interview).

“A very important problem is that people come here for someone to listen to them. They don’t have anyone else to talk to.”

(Focus group with healthcare professionals/Nuestra Sª del Pilar HC Center).

### 3.2. Obligations of Daily Life and the Perception of Pain

This category reflects the daily obligations perceived as undesirable by the CNP patients yet they felt obligated to fulfill due to duty or external pressures. These obligations often disrupt personal balance, potentially influencing pain perception. For most women, responsibilities such as caring for family members (including spouses, children, parents, and grandchildren), household chores, and tending to ill relatives were obligations that were not always desired. Conversely, for men, these obligations revolved mainly around their jobs and economic issues.

“While I am working, children make me feel agitated every day. Then I get home, and my mother is there, and I must take care of her. I’ve spent at least four years taking care of her, nonstop, and with all my energy.”

(Participant 3/1st interview).

“Everything piles up for me, daily, I have a hard job, my mother is old, and I must take care of her, and I have a daughter with Asperger’s, and you do not stop, you are under stress all day long. I am in tension all day long.”

(Focus group with patients/Juan de Austria HC Center).

“Maybe I’m doing something I should not be doing (at work), and it’s my fault, because there are things that I can refuse to do, but I don’t like to call and ask “hey, send me help…”, I can do it, but then I get angry with myself.”

(Participant 7/1st interview).

“And I work, and I don’t stop, and I try to do my best so that everyone is taken care of and happy…”

(Participant 9/1st interview).

### 3.3. Emotional Conflicts Related to CNP

This category encapsulates the conflictive situations within social relationships characterized by a significant emotional burden, complexity in resolution, difficulty in expression, and frequent recurrence in daily life. These conflicts frequently engender conflicting tendencies in individuals, leading to stress, discomfort, and emotional blockages. These experiences frequently trigger SP. It serves as the central category of the study, and comprises the following subcategories: Emotional strain resulting from conflict; Emotions during conflict; Bodily reactions to emotional strain; Individuals involved in conflict; Conflict resolution strategies.

#### 3.3.1. Emotional Strain Resulting from Conflict

The study participants expressed instances of conflict that highlighted a lack of self-respect and a devaluation of their own needs compared to those of others. Frequently, they opted to address these situations by prioritizing the needs of others, often at the expense of their own well-being. They avoided confrontation. Moreover, they expressed feelings of incomprehension, worry, and rumination underlying the conflicts.

“Nevertheless, I have a feeling of uneasiness inside, honestly … I prefer to lose this rather than spoil my relationship with a family member—I’m very clear about that. I prefer to lose out than end up with a sister-in-law who won’t talk to me.”

(Participant 2/1st interview).

“I always have to be supportive of him (husband) and take it easy, moving forward. Maybe I always put what’s important to me on the back burner.”

(Participant 9/1st interview).

“I feel cheated… With that person I feel cheated …, I feel resentful …, and I restrain myself.”

(Participant 11/1st interview).

“One of the things I see is a lot of worry, verbalization about their circumstances, a lot of self-examination… I see people who think too much, people who have obsessive thought, that’s what I find.”

(Focus group with healthcare professionals/Reyes Magos HC Center).

#### 3.3.2. Emotions During Conflict

The conflict-induced emotional strain elicited a variety of emotions and feelings among the participants. The most frequently cited emotions included anger, fear, and resignation. Additionally, the participants mentioned experiencing sadness, indignation, and frustration, followed by feelings of guilt, humiliation, and abandonment.

“That afternoon I had a very bad afternoon because my husband said that nothing was wrong, so I felt like crushing him at that moment, I was very angry and worried, honestly, and the pain increased, I endured it and then pain increased even more.”

(Participant 2/1st interview).

“And that fear I have … I always fear that something is going to happen at Christmas, like what happened in the past, and I get tense. Right now, I’m tense.”

(Participant 8/1st interview).

“I’ve lost all hope and I’ve already lost … I do not care anymore. I have given up. I don’t like it this way, and there’s nothing else.”

(Participant 4/1st interview).

#### 3.3.3. Bodily Reactions to Emotional Strain

As detailed in Section 2 (Materials and Methods), to explore the bodily responses to emotional stress triggered by interpersonal conflict, the participants were instructed to mentally relive personal conflict experiences and to engage with the associated emotions. Following this, they were asked to observe and to describe their bodily sensations, identifying the specific areas where these sensations manifested.

When mentally reliving the conflicts, most participants reported experiencing tension and/or pain, mainly in the cervical area, shoulders, head, and arms. Among male participants, tension was commonly reported in the trapezius, shoulders, and arms, resembling muscle activation associated with restrained physical aggression. By contrast, female participants often experienced greater tension in the neck and trapezius region, adopting a posture that suggested protective behavior by hunching their head between their shoulders. It is plausible that the observed divergences reflect differing coping styles rather than gender per se. Healthcare professionals have often noted that their patients somatized emotional tension by manifesting increased tension in the neck region. Furthermore, verbal expression and continuous activity emerged as additional methods used by the participants with CNP to relieve pent-up emotional tension.

“Well, a lot of tension is in the back. In all these areas of the shoulder blades and neck (the patient points to the back of the neck, left side and left shoulder), my stomach also gets upset.”

(Participant 2/2nd interview).

“Mostly tension in the arms … to be able to lift him up and… Same as a boxer, to lift him up and twist his neck.”

(Participant 12/1st interview).

“I’m tense. It happens to me a lot, I have tension in my shoulders when I’m going to talk (about the conflict), and they tell me, don’t get tense, and I don’t realize it, when I talk, I become tense.”

(Focus group with patients/Reyes Magos HC Center).

“In addition, in my patients these cervical pains usually appear at a specific point in time, the symptoms coincide when there has been something unpleasant, the children have left, the husband is not around…, something has happened.”

(Focus group with healthcare professionals/Reyes Magos HC Center).

#### 3.3.4. Individuals Involved in Conflict

Close relations, such as spouses, children, co-workers, and friends, were often involved in conflicts, triggering a range of emotions and stress responses.

“He’s my partner, he doesn’t believe in depression, he doesn’t believe in that, so he can’t help me. If something happens to me, well, yes, he suffers, but he doesn’t understand what’s happening to me.”

(Participant 1/1st interview).

“They don’t understand this. My children say that I am to blame for the entire current situation, yes, the blame… Now they no longer understand me… My children! They told me things that had never been said to me before, besides addressing me with contempt and things like that.”

(Participant 6/1st interview).

#### 3.3.5. Conflict Resolution Strategies

In the second interview, some CNP patients found resolution by reinterpreting their conflicts internally through increased self-respect and self-appreciation, which improved their communication and ultimately alleviated emotional strain and pain perception. These changes were facilitated by the emotional support they received.

“I felt like I put myself down, as if I wasn’t worth anything, as if people were attacking me because I was asking for it … but now, I’m really starting to see my life as it was before those problems. I have to overcome this, I have to appreciate myself, I have to value myself more than I did before… I feel capable, I can take on the world now … valuable, I value myself now more than ever.”

(Participant 6/2nd interview).

“Now as a woman I feel very complete, very much valued, starting with myself, of course. And I see that there are many people who love me. I feel much better.”

(Participant 8/2nd interview).

In summary, interpersonal conflicts and the resulting emotional strain appear to significantly influence the pain experience of the participants with CNP in this study. Prioritizing others’ needs over their own, coupled with self-devaluation, often leads to unresolved emotional strain and an increased perception of pain. Resolution involves internal shifts towards self-respect, self-esteem, and effective communication. Table 2 provides a summary of the themes, systematically organized into categories, subcategories, and corresponding codes.

## 4. Discussion

The findings from this study, based on the perspectives of the participants with CNP and the healthcare professionals who care for them—analyzed through a phenomenological lens—help highlight the role emotional conflicts may play in pain perception. Throughout the interpretive process, the researcher’s situatedness was acknowledged as part of the analytical lens. Rather than being seen as a limitation, this perspective was considered a potential source of depth and insight, helping to shape a more meaningful understanding of the participants’ experiences. Reflexivity remained central to the research process, supporting transparency and encouraging a critical awareness of how the interpretations were formed. The practice of dwelling with the data, alongside ongoing discussions within the interpretive team, contributed to a nuanced and thoughtful engagement with the narratives, ultimately strengthening the credibility of the analysis.

The participants’ narratives suggest that unresolved emotional conflicts may manifest as an intensified perception of CNP. This perception could be shaped by sustained muscular tension [48,54] and the physiological stress responses elicited by such conflicts [55]. At the same time, the persistent nature of CNP could impede the resolution of emotional tensions by disrupting cognitive clarity, emotional regulation, and habitual behavioral responses [56,57]. This interpretation underscores the intertwined nature of emotional and bodily experience, as conveyed by those living with CNP.

### 4.1. Self-Concept and the Experience of Pain

An individual’s self-concept influences the perception of his or her social roles, worldview, and behavior. Personal beliefs and values play a crucial role in shaping how social situations are interpreted and how interpersonal relationships are managed. As shown in this study, certain social contexts may be perceived as conflictual by individuals with CNP, which could elicit stress and SP responses that, in turn, may modulate their overall pain perception [37]. SP activates the brain networks linked to the affective dimension of pain [36].

In addition, interpersonal–emotional conflicts may also reinforce feelings of helplessness, undermining the self-concept [7]. And SP has been shown to reduce self-esteem, increase aggression toward others, and impair effective coping mechanisms for pain [58].

The aforementioned findings appear to close a vicious circle in which self-devaluation may play a substantial role that relates to the perception of CNP. Feelings of self-devaluation, commonly reported among the participants, may undermine the individuals’ self-confidence. Self-confidence and self-efficacy are pivotal in adapting to physical pain and SP, as they mitigate fears, foster perseverance, and enhance the likelihood of success [59]. Helplessness beliefs are associated with catastrophizing and heightened perception of pain intensity [60], while levels of self-esteem are significantly lower among individuals prone to pain compared to their healthy counterparts [61]. When confidence in one’s own coping abilities is lacking, individuals may resort to passive coping strategies, exacerbating perceptions of pain, disability, and depression [59].

Furthermore, the need for expression emerges as a prevalent personal trait among the participants. This could be explained by the fact that communicating concerns appears to diminish obsessive ruminations over time and to enhance physiological activity [62,63]. Conversely, a lack of social connections heightens the risk of depressive episodes in vulnerable individuals [64]. The evidence underscores the beneficial impact of trusting others in depression prevention, emphasizing the protective role of social connections against depressive symptoms [65]. And depression is related to the perception of CNP [46].

### 4.2. Obligations of Daily Life and Pain Perception

The participants in this study expressed discontent with numerous daily obligations and felt compelled to fulfill them, often resulting in stress. The participants’ self-devaluation could lead them to accept undesirable tasks or tasks that are beyond their capabilities, thus becoming stressors that endanger their well-being. Over time, individuals may exceed their adaptive capacities, which could hypothetically lead to the development of general adaptation syndrome. This prolonged neurobehavioral response is known to have detrimental effects on health [66], and contributes to an increased perception of pain [42,43,67].

### 4.3. Emotional Conflicts Related to CNP

Based on the participants’ experiences, conflicts that impact interpersonal relationships, recurring in daily life, proving complex to resolve and challenging to verbalize, elicit emotional strain and SP. In this sense, several studies have demonstrated that conflict and overwhelming circumstances act as stressors, and general adaptation syndrome could be triggered [42,43,67,68,69]. However, this reaction may or may not reach the consciousness [69]. The prominent factor in determining distress seems to be the disruption of social relationships, surpassing the impact of overall physical impairment [70].

In conflictive situations, the interviewed subjects often experienced internal contradiction, weighing the options of self-satisfaction versus complying with the demands of others. Frequently, they opted for the latter, possibly to avoid confrontation. Choosing to comply with others may serve to minimize unpleasant emotions, SP, and consequently physical pain. Individuals tend to avoid situations where rejection is anticipated, as it could be emotionally painful [37,70]. Conflict could act as a catalyst for learning strategies aimed at avoiding harm [71]. And the inclination towards harm avoidance could be linked to a less efficient conditioned pain modulation response, explained by mechanisms involving neurotransmitters and similar brain regions [72].

The participants frequently mentioned experiencing incomprehension. Feeling in-comprehension is known to make it difficult to seek validation for one’s pain in social circles and to access support as a coping strategy, which is associated with less positive functional outcomes in people with CP [73,74,75]. In addition, increased social disconnection during social interactions correlates with heightened activity in the dorsal anterior cingulate cortex in response to social exclusion, which in turn influences physical pain [76].

The participants also often express worry regarding the conflict. Worry has been shown to be a general vulnerability factor to psychological stress in adults [77]. It is worth noting that sensitivity to psychological distress parallels that of physical pain [37]. In addition, it is plausible that worry and conflict rumination may facilitate the individual’s repeated re-experiencing of SP. In this regard, studies have shown that remembering past SP is more likely to cause greater pain in the present, and imagining future SP also causes more pain in the present [40,41]. In both cases, the connectivity of the affective pain system could be activated, which depends on the dorsal prefrontal cortex to internally amplify the PS [78].

The participants expressed a variety of emotions in relation to the conflict, primarily anger, fear, and resignation. These conscious and/or unconscious emotional reactions to perceived circumstances may shape the neurophysiological processing of stress, SP, and consequently CP [7]. The specific emotions generated depend on the mood experienced, and could have a significant impact on conflict behavior [79].

In the present study, most of the interviewed women showed a need to verbally ex-press their emotions but often found themselves unable to do so. Conversely, most of the interviewed men felt the need to externalize their emotional strain through action but inhibited themselves due to societal norms and values. Although these distinct patterns of tension and pain have been interpreted as gender-related, the notably low proportion of male participants in this study limits the extent to which these findings can be attributed to gender differences alone. These differences could reflect distinct coping mechanisms rather than being inherently linked to gender. The participants in this study, both men and women, appear to avoid confrontation and feel misunderstood. Actively avoiding thoughts and feelings, or not expressing them, represents a particularly insidious form of inhibition. In the short term, inhibition could lead to increased activity of the autonomic nervous system, and over time, it could act as a cumulative stressor that raises the risk of somatic illness [80]. Moreover, feelings of incomprehension may foster insecure attachment, which in turn anticipates greater catastrophizing in response to pain [81].

On the other hand, and considering the Gray’s Reinforcement Sensitivity Theory, the inhibition responses of the participants could be related to the activation of the behavioral inhibition system in response to cues that signal potential punishment and promote withdrawal responses. The behavioral inhibition system activation has been significantly and positively associated with pain catastrophizing, anxiety, depression, and pain interference, and negatively associated with activity participation, hope, and pain self-efficacy [82].

Subjects engaged in interpersonal emotional conflicts often involve individuals close to the participants. The feeling of rejection or loss of people close to the subject could trigger SP, which is reflected in the perception of physical pain [35,37]. Furthermore, the evidence suggests that relationships with individuals close to the subject with CP may be stressful, particularly if they exhibit overprotective or excessively critical behavior. Such relationships have been associated with increased pain, stress, and disability [83].

The solution to daily interpersonal conflicts appears to involve reinforcing self-respect, self-esteem, and communication. Several participants were able to resolve their conflicts, resulting in a decrease in pain, as they themselves expressed. They attribute this achievement to the increase in self-confidence and the improvement in their self-concept, which they attained through emotional and social support. This support appears to help individuals overcome their fears and move beyond their comfort zone [84]. Emotional support has been shown to increase activity in the medial and left lateral prefrontal cortex, as well as several temporal regions. This suggests that emotional support may lead to a reduced affective response to pain [85].

In the present study, the sample includes a high proportion of participants with lower educational attainment. This factor may have influenced the results, as previous research has shown that individuals with lower levels of education—particularly those without university degrees—have significantly higher odds of experiencing chronic musculoskeletal pain. This has been linked to reduced access to healthcare resources and less effective pain management strategies compared to those with higher education levels [86,87,88,89]. The way our participants perceive their pain, particularly its intensity, may therefore be shaped, at least in part, by their educational background.

Moreover, the sample in this study is predominantly composed of women, reflecting a proportion similar to that reported in the scientific literature [86]. This high proportion of female participants may have influenced the findings. Although, in clinical practice, the interpretation of the Numerical Rating Scale as indicating mild, moderate, or severe pain in terms of functional interference is generally independent of the patient’s sex, it appears to be influenced by the individual’s tendency to catastrophize [90]. Since pain catastrophizing has been shown to significantly affect pain perception, the predominance of women in the sample—who tend to score higher on catastrophizing measures—could have impacted the reported pain experience [91].

To conclude, one could hypothesize that, when participants with CNP experience low self-esteem, feel misunderstood, or worry excessively, they may develop feelings of helplessness and respond with increased vigilance and harm avoidance. These strategies, in turn, may reinforce the same feelings of low self-esteem, misunderstanding, and worry. Avoidance-based coping mechanisms are known to negatively affect pain perception, disability, and depression in patients with CP [92].

### 4.4. Limitations of This Study

There are several limitations to this study. Firstly, no direct information was collected on the factors relevant to the interpretation of the results, such as alexithymia, self-esteem, empathic capacity, history of previous trauma, attachment style, stress levels, expectations, catastrophizing, or coping styles. Another limitation that may have influenced the findings is the predominance of female participants. Pain perception, conflict interpretation, coping styles, and other psychological and behavioral factors may be influenced by gender-related variables. In this study, 75.6% of the participants were women, which could introduce a gender bias in the results. Furthermore, this study was conducted in only one location, specifically in Primary Health Care settings, and focused solely on CNP. These limitations may restrict the interpretation of the findings and the depth of understanding of the phenomenon.

Future research should aim to address these limitations by investigating additional types of CP beyond CNP. These studies should consider the above-mentioned psychosocial factors and coping mechanisms when interpreting the results. Additionally, research is needed in diverse settings and populations to improve understanding of this phenomenon and to provide knowledge that will enable personalized interventions for the effective treatment of CP.

### 4.5. Implications for Practice and Future Research Directions

Based on the results, and to provide effective and comprehensive care, it is essential that health professionals consider the following:

- Understand the relationship between physical pain and SP.
- Consider the potential influence of the patient’s interpersonal conflicts on pain perception, including present and past conflicts that the patient may mentally relive or project into the future, particularly if these conflicts are repeated and persist over time.
- Actively inquire about interpersonal conflicts, especially if the patient hints at them or if suspicions arise from her speech.
- Provide assistance to patients experiencing SP or refer them to an appropriate professional for further support.

## 5. Conclusions

The perspectives of the participants with CNP in this study underscore the perceived impact of interpersonal conflicts on their pain experience using hermeneutic phenomenology. Stress and emotional tension arising from such conflicts were reported to potentially intensify pain and physical symptoms, particularly in the cervical region, head, shoulders, and arms. Both the direct experience of conflict and the mental reactivation of these experiences appeared to contribute to heightened pain perception. Recognizing the interplay between interpersonal conflict, pain, and physical discomfort may be important in developing more comprehensive care approaches for individuals with CNP. The findings of this study may assist physiotherapists in grounding their educational strategies within CP treatment. Furthermore, they could contribute to the design of interdisciplinary therapeutic programs aimed at a more comprehensive approach to CP management.

Identify multidimensional aspects of chronic pain, including its characteristics and quality, associated symptoms, and psychological factors such as fears and worries. Explore the pain’s impact on daily activities, behavioral and mobility changes, factors influencing pain, patient expectations, and their perceived role in treatment and pain progression.

Explore the clinical and behavioral features of the patients with non-specific chronic neck pain, the diagnostic and management approaches in primary care, and the challenges encountered. Examine patient coping strategies and their effectiveness, as well as perceptions of the patients’ roles in their treatment and pain progression.

## Figures and Tables

**Table 1:** Sociodemographic and clinical data of the participants.

Participants	Sociodemographic and Clinical Data		
Participants with non-specific chronic neck pain (N = 23)	Age	Years M (SD)	53.9 (14.4)
	Sex	Women N (%)	31 (75.6%)
		Men N (%)	10 (24.4%)
	Study level	No studies N (%)	3 (7.3%)
		Primary studies N (%)	21 (51.2%)
		High school studies N (%)	14 (34.2%)
		University studies N (%)	3 (7.3%)
	Employment status	Active N (%)	19 (46.3%)
		Retired N (%)	10 (24.4%)
		Housewife N (%)	12 (29.3%)
	Goldberg Anxiety and Depression Scale	Likely anxiety N (%)	29 (70.7%)
		Likely depression N (%)	30 (73.2%)
	Pain intensity	Visual Analogical Scale (mm) M (SD)	48.1 (20.7)
Healthcare professionals (N = 46)	Profession	Physicians N (%)	20 (43.5%)
		Nurses N (%)	22 (47.8%)
		Physical therapists N (%)	4 (8.7%)

N: number of subjects; % percentage; M: mean; SD: standard deviation.

**Table 2:** Categories, subcategories and codes.

Primary Categories	Subcategories	Codes
Self-concept and pain experience	Beliefs about the self	Nervous
		Perfectionists
		Having communication issues
	Personal values	Family
		Work
		Sexuality
	Personality traits	Self-devaluation
		Need for expression
		Need for understanding
Daily life obligations and pain perception	Caring for family members	Caring for family members
	Household chores	Household chores
	Tending to the ill	Tending to the ill
	Job	Job
	Economic issues	Economic issues
Emotional conflicts related to CNP	Emotional Strain resulting from Conflict	Lack of self-respect
		Feelings of incomprehension
		Worry
		Rumination
		Lack of control
		Inability
	Emotions during Conflict	Anger
		Fear
		Resignation
		Abandonment
		Guilt
		Humiliation
	Bodily Reactions to Emotional Strain	Neck
		Shoulders
		Head
		Arms
		Stomach
	Individuals Involved in Conflict	Spouses
		Children
		Co-workers
		Friends
	Conflict Resolution Strategies	Self-respect
		Self-appreciation
		Improving communication

## Data Availability

The data presented in this study are available upon request from the corresponding author.

## Abbreviations

The following abbreviations are used in this manuscript:

CNP	Non-specific chronic neck pain
CP	Chronic pain
SP	Social pain
HC	Primary Health Care Center

## Appendix A

**Table A1:** Guide for the Semi-Structured First Interview.

1st Interview Stage	Target	Content
Start	Introductory question	Welcome and initial greeting. Brief explanation of what will be asked and its objective. Understand the individual’s current feelings and obtain a general overview of their experience with neck pain.
Development	The perception of pain and its impact	Describe the characteristics, intensity, and impact of neck pain in an individual.
	Stressors related to pain	Explore the presence and sources of stress related to occupational and daily responsibilities, including feelings of being overwhelmed and the impact of interpersonal conflicts.
	Self-perception	Explore self-identity, personality, and values in the context of chronic pain.
	Emotional conflicts and pain perception	Explore the relationship between daily conflicts or stressful situations and exacerbation of neck pain. Identify specific triggers, emotional responses during such events, and conflict management strategies.
	Bodily sensations related to conflicts	Investigate the bodily sensations associated with recalling interpersonal conflicts by guiding the participants to mentally relive these situations, with a focus on the location, quality, and intensity of the somatic experiences.
Closing	Final input and acknowledgments	Offer the interviewee the opportunity to add any further information. Thank them for their contribution.

**Table A2:** Guide for the Semi-Structured Second Interview.

2nd Interview Stage	Target	Content
Start	Introductory question	Welcome and initial greeting. Understand the individual’s current feelings. Obtain a general overview of their experience with neck pain following a physiotherapy treatment.
Development	Current pain status	Monitor the progression of neck pain over time by assessing the changes since the previous interview.
	Current impact of pain on daily life	Assess the impact of pain-related changes on daily activities and lifestyle. Explore which specific aspects have been affected. Investigate the emotional responses associated with these changes.
	Current identification of pain sources and underlying causes	Explore significant life changes since the previous assessment that may influence the individual’s pain experience. Identify the individual’s current primary concerns that could be contributing to or exacerbating their pain.
	Follow-up on emotional conflict and its impact on current pain perception	Evaluate whether the previously discussed conflict remains unresolved or has been resolved. Examine how either condition affects the individual’s pain. Explore the impact of conflict resolution, if applicable, on pain levels and the strategies employed to address the conflict.
Closing	Final input and acknowledgments	Offer the interviewee the opportunity to add any further information. Thank them for their contribution.

**Table A3:** Guide for the Focus Groups with the Patients Experiencing Non-Specific Chronic Neck Pain.

Stage	Target	Content
Start	Introductory question	Welcome to the participants. Brief explanation of how the session will be conducted.
Development	Symptoms and relevant aspects in the experience of pain	Identify multidimensional aspects of chronic pain, including its characteristics and quality, associated symptoms, and psychological factors such as fears and worries. Explore the pain’s impact on daily activities, behavioral and mobility changes, factors influencing pain, patient expectations, and their perceived role in treatment and pain progression.
	Pain-related stressors and their impact on pain perception	Examine the stressors that exacerbate pain, the influence of interpersonal relationships on pain perception, and the dynamic interaction between emotional and physical pain.
Closing	Final input and acknowledgments	Offer the participants the opportunity to add any further information. Thank them for their contribution.

**Table A4:** Guide for the Focus Groups with the Healthcare Professionals.

Stage	Target	Content
Start	Introductory question	Welcome to the participants. Brief explanation of how the session will be conducted.
Development	Non-specific chronic neck pain	Explore the clinical and behavioral features of the patients with non-specific chronic neck pain, the diagnostic and management approaches in primary care, and the challenges encountered. Examine patient coping strategies and their effectiveness, as well as perceptions of the patients’ roles in their treatment and pain progression.
	Pain-related stressors and their impact on pain perception	Identify the stressors commonly linked to chronic neck pain and their clinical effects. Explore how the patients’ beliefs, emotions, and expectations shape their pain experience and treatment adherence. Discuss the interaction between emotional and physical pain.
	Professional experience about chronic pain	Address their personal feelings, beliefs, and expectations related to pain. Explore the challenges in managing the psychological and emotional aspects of pain. Identify the communication strategies employed with the patients. Assess the perceived need for further learning in pain management.
Closing	Final input and acknowledgments	Offer the participants the opportunity to add any further information. Thank them for their contribution.
